# Constructing a simplified interurban road network based on crowdsourced geodata

**DOI:** 10.1016/j.mex.2022.101845

**Published:** 2022-09-06

**Authors:** Rafael Prieto-Curiel, Inhoi Heo, Abel Schumann, Philipp Heinrigs

**Affiliations:** a Complexity Science Hub Vienna, Josefstädter Str. 39, Vienna 1080, Austria; b OECD Sahel and West Africa Club Secretariat, 2 rue André-Pascal, Paris 75775 CEDEX 16, France

**Keywords:** OpenStreetMap, Spatial network, Africa

## Abstract

The road network that connects cities with the existing road infrastructure of a country is a valuable tool for analyzing its transport routes, connectivity, and urban patterns. Yet, it is challenging to construct, given the data available.

• We present a method to construct a simplified and connected urban network. Some network nodes are cities, and others are “transport nodes” representing road crossings or other types of infrastructure.
• The result is a simplified connected network of all cities and the existing road infrastructure that maintains road distances and available routes.
• The procedure reduces millions of spatial points that sometimes are disconnected polygonal lines or patches into a connected network with only a few edges and nodes.

## Graphical abstract

**Figure:**
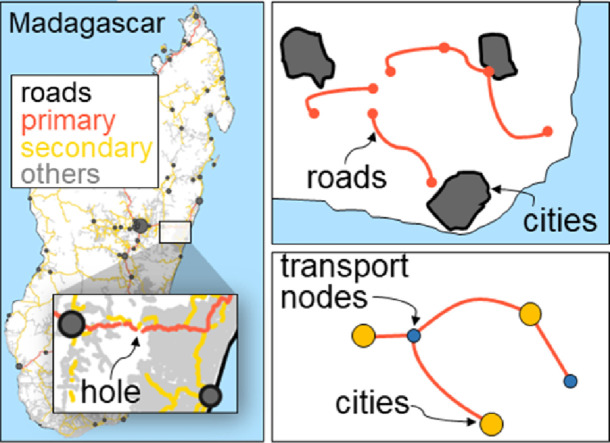

Specifications table

**Table:** 

Subject area:	
More specific subject area:	*Spatial networks*
Name of your method:	*Connecting a road network based on OpenStreetMap data*
Name and reference of original method:	*N.A.*
Resource availability:	https://github.com/rafaelprietocuriel/AfricanUrbanNetwork

## Method details

OpenStreetMap creates and distributes free geographic crowdsourced data [1]. Primary roads, highways and trunks can be obtained from OpenStreetMap to study the transport infrastructure of a country or a region. The data gives the x,y coordinates as a sequence of vertices of each road segment. However, the data is not perfect [2]. Take, for example, the road infrastructure of Madagascar, a country in East Africa with more than 28 million inhabitants in the fourth-largest island in the world. The raw data consists of 5.5 million vertices forming 164,540 road segments. A curved road is composed of a sequence of multiple vertices, so the length of a highway is approximated by the sum of all components, providing a reasonable estimate of road length. Thus, we can measure the physical distance between the two vertices as the length of that segment (using [3,4]) and have a good approximation of the road distance between any two locations in the network. With this input, it should be possible to detect the travel distance between any two cities and find the shortest route.

Many roads are interurban (mostly in Antananarivo, the country's capital), including streets and avenues within the main urban areas. To consider the infrastructure available in a country for interurban travel, we take into account only primary roads. Madagascar has 130,000 vertices arranged on 4,545 roads. Other road types, including paths and tertiary roads, are not considered since most are inside urban polygons and are not frequently used for intracity mobility.

**Fig. 1:**
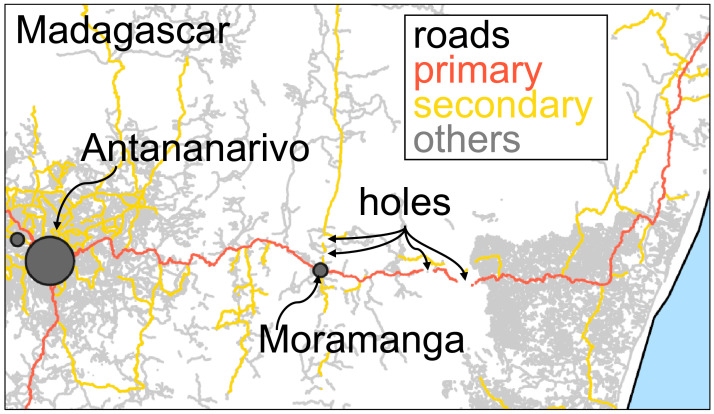
Primary, secondary, and other roads connecting Antananarivo to the East part of the country. The data was obtained from OpenStreetMap. The roads form disconnected segments even if different types of roads are considered.

With more than 4,500 roads in Madagascar, it is possible to travel between each pair of cities. But, mathematically speaking, is it possible to detect the paths? If each road starts where another road finishes, then the 4,500 roads define a continuous trajectory, from which we could define routes and compute distances between different origins and destinations. Unfortunately, there are some challenges in constructing the urban network. The road data is imperfect since it has “holes” (Fig. 1). For example, a road between two cities is often registered as many non-connected components. The road data initially forms many disconnected parts. This is a critical part since we always look for the shortest path between any two cities or any two locations in the network. And when the roads have holes, the shortest route that we find in the data might be substantially different and longer or might not even exist if the network is not connected. Thus, we must transform the original road data into a connected network. Also, in terms of size, Madagascar has only 26 different urban agglomerations [5], so the network dimension should not have thousands of edges but perhaps dozens. After all, any interurban journey should start at any 26 agglomerations and finish at any other 25.

Here, we present the steps we follow to start with the OpenStreetMap road infrastructure data, combine it with cities data and construct a connected and simplified network. The result is a network where most nodes are cities, and the remaining nodes are “transport nodes”, required to describe the road infrastructure within a network consisting of road crossings and where the edges are existing road infrastructure. We connect cities and highways based on proximity. This means that if the ending of one road is sufficiently close to the beginning of another, we assume that it is possible to travel between them. Also, if a city is sufficiently close to a road, we assume that the people of that city can use that road.

Our process depends on parameters, such as defining how “close” a road must be to another to be considered connected roads. There are two possible errors with our procedure. One type of error is connecting parts where a road does not exist, and the second type is leaving two nodes disconnected where it is possible to travel between them. Since the objective is to measure distances, the second error is more critical. Network distances are highly sensitive to disconnections, so we try to keep this error to a minimum. Therefore, we assume that even if some of the roads are not present in the data, it is possible to travel between nodes if the distance between them is sufficiently small.

## Process

Our process consists of three sequential steps:

(1) Align nodes of interest (cities) and road infrastructure.
(2) Connect roads to each other based on their proximity.
(3) Simplify the network by dissolving nodes.

After the three steps, an easy-to-manipulate network is obtained.

**1. Align nodes of interest (cities) and road infrastructure**

Cities and roads are not spatially aligned, meaning that a road might pass close to an urban area to consider it part of the network. Thus, we begin the process by connecting cities to the primary road data. Here, if the distance between the centroid of a city and a transport node is less than C=10 km, we assume that the road passes through that city, so we label the closest transport node as a city node. Even if cities are not directly connected to the road, a 10 km threshold is still within a reasonable distance to gain from the infrastructure. We use C=10 km threshold since even smaller cities still gain from that road infrastructure. The corresponding city is used as the label of the road vertex. For Madagascar, 14 urban agglomerations are within 10 km of a road vertex (including all cities in the country with more than 80,000 inhabitants). Within those 14 urban agglomerations, we have 4.1 million people. Only 12 cities are not close enough to the road infrastructure, but they are very small agglomerations (in total 530,000 people, where the largest city has 70,000 inhabitants. This step does not alter the network structure, so it does not change distances between nodes. It only assigns labels to some of the road nodes.

We measure the degree of all transport nodes, $\delta_{k}=2$, given by the number of roads connecting it. The remaining vertices from the road infrastructure are labelled as terminal if it is starting or ending point of the road (if $\delta_{k}=1$), as crossing if there are more than two options on that node (if $\delta_{k}>2$), and simply as road nodes if it is neither. A first network is constructed using all the labelled vertices as nodes, and the corresponding road segments are the edges, using [6,4]. For each edge, we keep the type of road and its length. In Madagascar, this initial network has more than 45,000 nodes.

**2. Connect roads to each other based on their proximity**

The first step is to create a connected network. We explore all terminal nodes. If a terminal node is less than T=24 km away from any other road segment, we assume it is possible to travel between them, so we add that edge. Here, we use a threshold of T=24 km, assuming a person could use alternative methods to travel between the two terminal nodes if the infrastructure does not exist. Since that edge did not exist in the OpenStreetMap data, we labelled it “added”. Two terminal nodes might become road nodes if they get connected, but also, a road node might become a crossing if a new travel option is added. By the end of this procedure, the network has no terminal nodes at a short distance from others.

Second, we check whether there are any separate components in the graph. We identify the physical distance between all the nodes in one of the components against every node in the remaining part of the graph. We add the corresponding edge with the shortest distance and label it “added”. We cannot guarantee that travelling through them is feasible or efficient (fast) for all the added edges, but the result is a connected network. We can then estimate the network's travel distance between all pairs of nodes and whether the edges are primary roads, highways or if our procedure added them.

Finally, for every pair of nodes with a physical distance smaller than T=24 km, we compute their road distance (the sum of the lengths of all the shortest path segments) and compare it against its physical distance. If the ratio exceeds 10 (meaning that travelling through the network is ten or more times longer than travelling through the geodesic that connects them), we add the edge between them and label it “added”. Again, in the worst-case scenario, it would be possible to travel between those two nodes using alternative methods rather than driving through within a ten or more-times longer road. We also consider pairs of cities and transport nodes at a distance smaller than T=24km and compare the network and spatial distances. If the ratio is greater than 10, we add that edge. Likely, these roads correspond to secondary roads or other types of infrastructure beyond highways. Although there might be some errors here, where connections that do not exist are introduced, it reduces a more severe issue: seemingly disconnected patches of nearby nodes.

**3. Simplify the network by dissolving nodes**

We simplify the network by removing (dissolving) road nodes with a degree $\delta_{k}=2$ connecting the same road type at both ends. We sum the lengths of the corresponding segments to form the new edge. We replace the road node with a longer edge. Each step drops one node and edge from the network without losing the details about distances. Also, we remove all loops, that is, edges that begin and end in the same node.

**Fig. 2:**
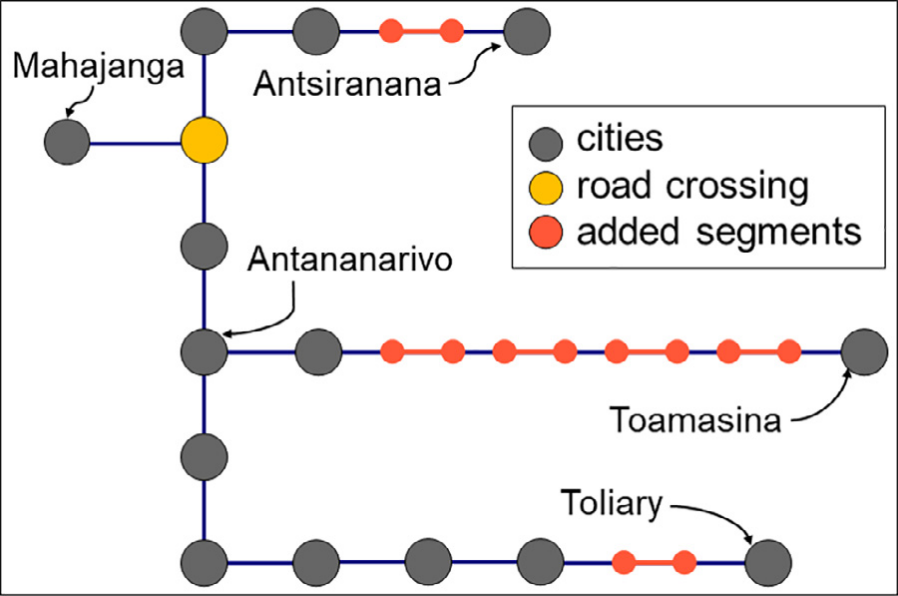
Simplified network of Madagascar with 14 cities and 13 transport nodes (one road crossing and 12 corresponding to changes between added and detected roads) and 26 edges. In total, 2600 km of road infrastructure are considered. Less than 50 km of roads are added to the network within the six added edges, less than 1.8% of the road infrastructure.

Data from OpenStreetMap in Madagascar contains 5.5 million vertices forming more than 160,000 road segments. After our procedure, we have obtained a network that describes the available infrastructure in Madagascar for intercity journeys. The network comprises 14 cities and 15 transport nodes, from which there is one road crossing and 14 additional nodes where the type of road switches (Fig 2). The network has 26 edges connecting it. For each edge, we estimate its road distance, so it is possible to detect the shortest path between all pairs of cities. The added segments represent less than 2% of the length of the roads in the country, but they enable us to obtain a connected network. The same procedure has been applied to data for continental African cities. The continental network has 7,361 nodes (2,162 are cities and 5,199 are transport nodes), and the network has 9,159 edges connecting its nodes. The network data is available at [7] and the code at [8]. It can be used to model interurban mobility patterns [9] and clustering of urban agglomerations [10], among many.

Our procedure can also be used for other types of infrastructure, although the parameters C=10 km and T=24 km should be adjusted. For example, a similar method could be applied for constructing the network of cycling roads in a city but with different parameters.

## Ethics statements

Our work does not involve animals or humans as subjects. All data used and produced in the manuscript is open access.

## CRediT authorship contribution statement

**Rafael Prieto-Curiel:** Writing – original draft, Methodology. **Inhoi Heo:** Methodology. **Abel Schumann:** Methodology. **Philipp Heinrigs:** Methodology.

## References

[1] OpenStreetMap contributors. Planet dump retrieved from https://planet.osm.org. https://www.openstreetmap.org, 2021.

[2] Yeboah G., Porto de Albuquerque J., Troilo R. (2021). Analysis of OpenStreetMap data quality at different stages of a participatory mapping process: evidence from slums in Africa and Asia. ISPRS Int. J. Geo Inf..

[3] Hijmans R (2021). geosphere: Spherical Trigonometry. R package version 1.5-14.

[4] R. Core Team, R: A Language and Environment for Statistical Computing. R Foundation for Statistical Computing, R Core Team, Vienna, Austria, 2022. https://www.R-project.org/.

[5] Moriconi-Ebrard, F. D. Harre, and P. Heinrigs, (2016). Urbanisation Dynamics in West Africa 19502010. OECD: Organisation for Economic Co-operation and Development.

[6] Csardi G., Nepusz T. (2006). The igraph software package for complex network research. Int. J. Complex Syst..

[7] R. Prieto-Curiel, A. Schumann, I. Heo, and P. Heinrigs, A dataset of the network of African cities and highways, Data Brief (2022).

[8] Prieto-Curiel, R. African Urban Network [Data set] https://github.com/rafaelprietocuriel/AfricanUrbanNetwork.

[9] Prieto-Curiel R., Schumann A., Heo I., Heinrigs P. (2022). Detecting cities with high intermediacy in the African urban network, Computers. Environment and Urban Systems.

[10] Prieto-Curiel R., Cabrera-Arnau C., Bishop S.R. (2022). Scaling beyond cities. Front. Phys..

